# Development and validation of a clinicopathological‐based nomogram to predict seeding risk after percutaneous thermal ablation of primary liver carcinoma

**DOI:** 10.1002/cam4.3250

**Published:** 2020-07-23

**Authors:** Chao An, Zhimei Huang, Jiayan Ni, Mengxuan Zuo, Yiquan Jiang, Tianqi Zhang, Jin‐Hua Huang

**Affiliations:** ^1^ Department of Minimal invasive intervention Sun Yat‐sen University Cancer Center Guangzhou China; ^2^ State Key Laboratory of Oncology in South China Guangzhou China; ^3^ Collaborative Innovation Center for Cancer Medicine Guangzhou China

**Keywords:** nomogram, primary liver carcinoma, risk analysis, seeding, thermal ablation

## Abstract

**Objectives:**

To develop a clinicopathological‐based nomogram to improve the prediction of the seeding risk of after percutaneous thermal ablation (PTA) in primary liver carcinoma (PLC).

**Methods:**

A total of 2030 patients with PLC who underwent PTA were included between April 2009 and December 2018. The patients were grouped into a training dataset (n = 1024) and an external validation dataset (n = 1006). Baseline characteristics were collected to identify the risk factors of seeding after PTA. The multivariate Cox proportional hazards model based on the risk factors was used to develop the nomogram, which was used for assessment for its predictive accuracy using mainly the Harrell's C‐index and receiver operating characteristic curve (AUC).

**Results:**

The median follow‐up time was 30.3 months (range, 3.2‐115.7 months). The seeding risk was 0.89% per tumor and 1.5% per patient in the training set. The nomogram was developed based on tumor size, subcapsular, α‐fetoprotein (AFP), and international normalized ratio (INR). The 1‐, 2‐, and 3‐year cumulative seeding rates were 0.1%, 0.7% and 1.2% in the low‐risk group, and 1.7%, 6.3% and 6.3% in the high‐risk group, respectively, showing significant statistical difference (*P* < .001). The nomogram had good calibration and discriminatory abilities in the training set, with C‐indexes of 0.722 (95% confidence interval [CI]: 0.661, 0.883) and AUC of 0.850 (95% CI: 0.767, 0.934). External validation with 1000 bootstrapped sample sets showed a good C‐index of 0.706 (95% CI: 0.546, 0.866) and AUC of 0.736 (95% CI: 0. 646, 0.827).

**Conclusions:**

The clinicopathological‐based nomogram could be used to quantify the probability of seeding risk after PTA in PLC.

## Graphical abstract

Seeding on the thoracoabdominal wall from primary liver carcinoma is a potential complication after percutaneous thermal ablation. Seeding risk is a liver ablation complication that affects future oncology management. A calibrated and objective model to predict seeding risk after liver ablation may guide patient selection and ablation treatment.

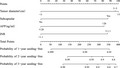

## 1 INTRODUCTION

Primary liver carcinoma (PLC) is the sixth most common malignant disorder and the third most common cause of cancer death global, which is caused commonly by altered expression of oncogenes and tumor suppressor genes.^1^, ^2^, ^3^, ^4^ Previous studies have reported in‐silico techniques related to oncogens.^5^, ^6^ Image‐guided percutaneous thermal ablation (PTA) is an acceptable and effective alternative with the advantages of shorter recovery time and minimally invasive, which is used in clinic widely.^7^, ^8^, ^9^ Various complications related to PTA have been reported. However, seeding from PLC on the thoracoabdominal wall is the most unfavorable complication, which occasionally emerges after the percutaneous procedures or surgery.^10^, ^11^, ^12^ Recently, risk of seeding after percutaneous ethanol injection (PEI), radiofrequency ablation (RFA), microwave ablation (MWA) and cryoablation varied from 0.3% to 1.9%, from 0.005% to 12.5%, from 0.44% to 0.75% and 0.76% in previous reports respectively, has received increased attention from clinicians.^13^, ^14^, ^15^, ^16^, ^17^, ^18^

Based on appropriate choice by the patient and careful attention to the ablation procedure, several studies showed low seeding rate after ablation. Nevertheless, even if the ablation modalities have been proven to be a relatively safe technique, seeding remains an inevitable ablation‐related complication. The occurrence of seeding has to be paid major attention, because the metastatic lesions may cause subsequent treatment to become complicated and difficulty. Most studies show risk factors of seeding after percutaneous ablation, mainly including tumor size, subcapsular location, biopsy preablation, ablation sessions, poor pathological differentiation and so on.^19^, ^20^ However, the conclusions of complete consistency remain fail to achieve in the large numbers of studies.

Nomogram is a visual risk regression model and an ideal tool for predicting patients’ prognosis, derived from various hazard functions. Therefore, nomogram was applied to predict the survival outcome of the patients liver cancer after surgical resection, transarterial chemoembolization (TACE) or radiofrequency ablation (RFA) and satisfactory ability in risk prediction.^21^, ^22^, ^23^ However, there has been a paucity of reports on nomogram for predicting the occurrence of complications, especially the risk of seeding after thermal ablation. Using nomogram can help physicians making preoperative planning before MWA to reduce seeding risk.

In this study, we developed a nomogram model to predict individual seeding risk after thermal ablation of PLC, with an aim to evaluate the impact of risk factors on the potential curative intent of seeding.

## 2 MATERIALS AND METHODS

### 2.1 Patient selection

**FIGURE 1:**
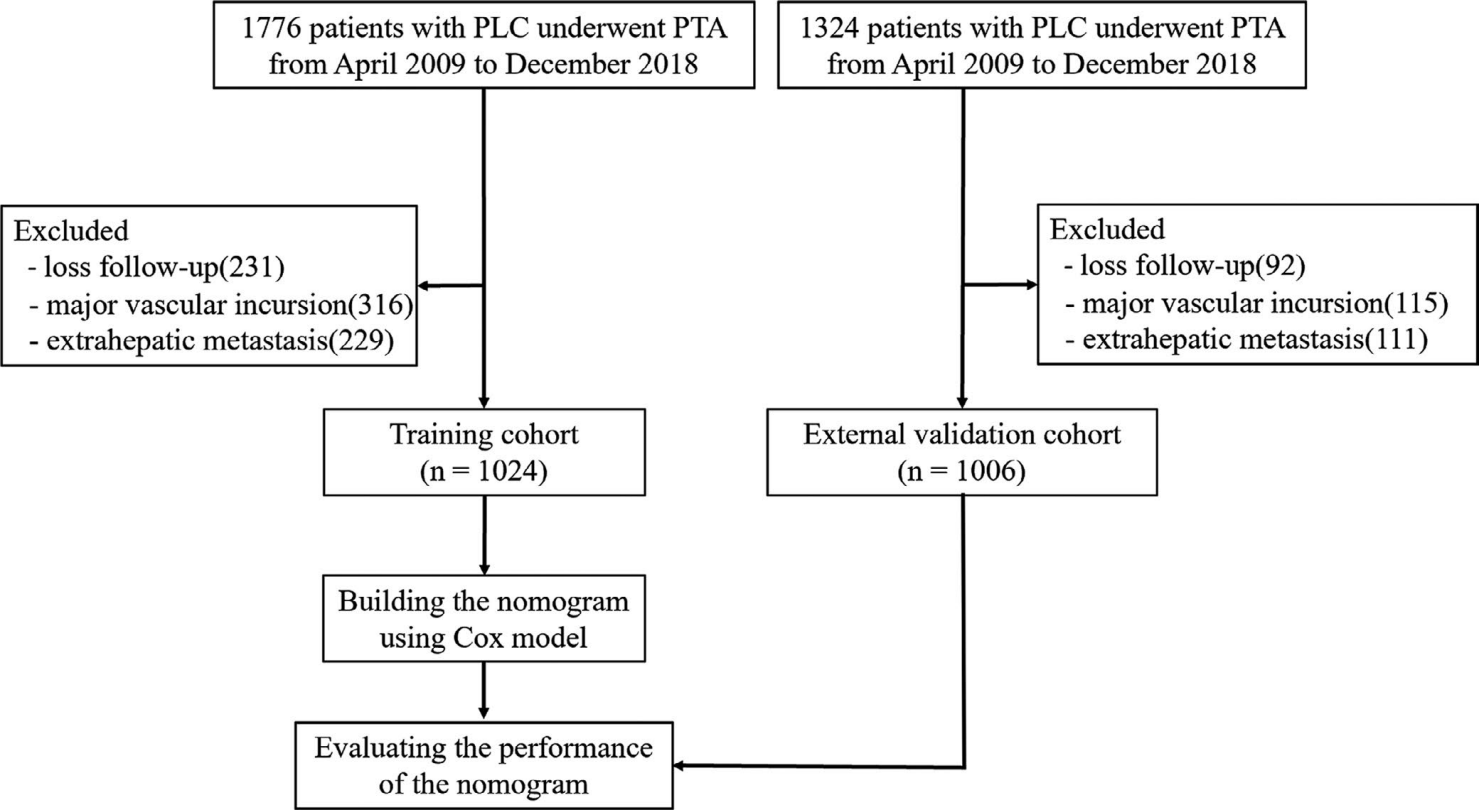
Flow diagram shows study patient accrual process

Protocols used at the two medical centers in this retrospective study received approval from the Ethics Committee and informed consent from patients was waived. Data on 1024 consecutive patients with primary liver carcinoma (PLC) (1670 nodules) who had undergone percutaneous thermal ablation (PTA) through MWA and RFA between April 2009 to December 2018 were reviewed. Data on an external validation set of 1006 consecutive patients with PLC (1578 nodules) who had undergone PTA during the same study period were also collected. PLC was diagnosed based the on American Association for the Study of Liver Disease (AASLD) and European Association for the Study of Liver (EASL)^24^, ^25^ guidelines and was confirmed using pathological findings from needle biopsy samples obtained before ablation. For this study, the inclusion criteria were: (a) Eastern Cooperative Oncology Group (ECOG) performance status of <2; (b) single tumors of not more than 12 cm in diameter and a total number of less than 3; (c) Child‐Turcotte‐Pugh (CTP) grade A or B. The exclusion criteria were: (a) loss during follow‐up; (b) extrahepatic metastasis; or (c) major vascular incursion. Figure 1 shows patient enrollment pathways and both inclusion and exclusion criterion.

### 2.2 PTA procedure

The device used has been described in detailed in a previous report.^26^ Before ablation, a 2‐cm skin incision was made using a scalpel, and 1% lidocaine (Yi you, Beijing, China) was subcutaneously injected as local anesthesia to moderately sedate each patient. An 18‐gauge cutting needle in an automatic biopsy gun was used to carry out the biopsy before thermal ablation. Consequently, an internally cooled, 17‐gauge, 15 cm, electrode, with a 2‐ or 3‐cm long exposed metallic tip (Cool‐tip; Covidien) or a 15‐gauge antenna (KY‐2000, Kangyou Medical) was percutaneously inserted into the tumor as preoperatively planned using US or CT guidance. The RF energy was delivered for 12‐16 minutes at impedance control mode, while for MWA, a power output of 50 W for 10 minutes was routinely used. The electrode or antenna was inserted repeatedly after minor changes were made to the tip angle, in order for the ablation zone to completely cover the tumor area with a safety margin of >0.5 cm. Finally, the MWA or RFA needle tract was cauterized during needle withdrawal.

### 2.3 Seeding and data analysis

Seeding was defined as the presence of a neoplastic nodule along the needle track outside the liver parenchyma, which invariably presented with single or multiple rounded nodules located on the peritoneum, thoracoabdominal wall muscles or subcutaneous tissues. Biopsy confirmation was not essential for the diagnosis. Suspicious cases were identified by review of all radiological and pathological records after biopsy or ablation. We showed two cases of seeding caused by RFA or MWA for HCC (Figure S1). Seeding may grow from 6 to 46 months after ablation using various modalities. The literature review conducted is summarized in Table S1. Tumors were defined as subcapsular when they abutted the liver surface and the shortest distance between the nodule and liver capsule was less than 5 mm. The clinical information and oncological outcomes related to seeding after PTA were collected, which included sex, age, tumor type, hepatitis type, differentiated degree, seeding location, seeding time, treatment modality, and overall survival (OS).

### 2.4 Follow‐up and assessment

Contrast‐enhanced multiphase images were obtained three days after the last course of a defined ablation period using computed tomography (CT) or magnetic resonance imaging (MRI) to assess the efficacy of treatment. Inadequate ablation was detected based on the development of asymmetrically dispersed, nodular or unusual patterns, and signified the need for another ablation. Comprehensive local necrosis of the target zone 1 month after PTA was used to indicate the effectiveness of the technique.^27^ Based on adequate ablation, 1 and 3 months after PTA and at intervals between 3 and 6 months serum, AFP levels and c multiphase images were obtained. The period from the date of the first session of PTA for liver malignancy to the last date of follow‐up (survival or loss) or date of death was used as the OS. Patients with seeding were treated with surgical resection, MWA or RFA, based on patient preference and multidisciplinary discussions.

### 2.5 Statistical analysis

The emergence of seeding after PTA was the outcome investigated in this study. Statistical analyses were conducted using the RMS package of R software version 3.6.1 (http://www.r‐project.org/) and SPSS 22.0 (SPSS) software. Continuous variables that fulfilled the normality assumption were compared using two samples t test, while the Mann‐Whitney *U* test was performed on nonnormally distributed continuous variables. Chi‐square test or Fisher's exact test was used as appropriate to analyze categorical variables. Tumor seeding rate was evaluated using the Kaplan‐Meier method with log‐rank test. Independent seeding risk factors were evaluated through univariate and multivariate analyses using the forward stepwise Cox regression model. Nomogram construction used Cox‐model derived β coefficients to evaluate the association between seeding risk and selected variables. An established nomogram was used to calculate the score for each of the validation group patients for external nomogram validation. All P values given are two‐sided and statistical significance was indicated by a *P* value of <.05.

## 3 RESULTS

### 3.1 Baseline characteristics

Baseline characteristics of patients with PLC (hepatocellular carcinoma (HCC) in 2000 cases and intrahepatic cholangiocarcinoma (ICC) in 30 cases) after PTA (MWA for 1701 cases and RFA for 329 cases) in the training set and validation set are shown in Table 1. No significant differences were found between the clinical characteristics and follow‐up data of both sets (*P* = .824‐0.867). About 97.6% of the technique effectiveness rate of the training set was comparable to 97.9% of the technique effectiveness rate in the validation set (*P* = .762). Patients in the training set were followed‐up after PTA for a median of 30.3 months (range 3.2‐115.7 months). Seeding was found in 15 patients with 17 nodules (0.89% per tumor, 1.5% per patient). The baseline characteristics of patients with seeding after PTA are shown in Table 2. The tumor type of the seeding nodule was PLC in twelve cases and ICC in three other cases. The seeding nodules were found to have a mean size of 3.8 ± 1.7 cm (range, 2.0‐6.4 cm). Seeding was located on the chest wall in five cases and on the abdominal wall in twelve cases. All patients with seeding were successfully treated using resection (two), MWA (twelve) and RFA (three), according to the size of the seeding nodules and preference of patients. Five patients with seeding had died as a result of PLC progression at the end of the follow‐up period and the median OS was 44.7 months (range, 14.7‐74.2 months).

**TABLE 1:** Baseline patient characteristics

Variables	Training set (n = 1024)	Validation set (n = 1006)	*P* value
Mean age ± SD (y) (range)	58.3 ± 11.2 (24‐91)	58.3 ± 10.9 (23‐86)	.824^a^
Sex			
Male	837 (78.5)	808 (82.5)	.139^b^
Female	187 (21.5)	198 (17.5)	
Performance status			
0	913 (95.0)	879 (94.7)	.898^b^
1	111 (5.0)	127 (5.3)	
Comorbidities			
Absence	410 (14.6)	48 (13.1)	.519^b^
Presence	614 (85.4)	318 (86.9)	
Etiology			
HBV positive	645 (78.3)	632 (78.4)	.333^b^
Other	379 (11.1)	374 (8.2)	
Cirrhosis			
Absence	94 (9.3)	89 (7.9)	.484^b^
Presence	930 (90.7)	917 (92.1)	
CTP grade			
A	976 (95.3)	967 (96.1)	.648^b^
B	48 (4.7)	39 (3.9)	
Median AFP level (ng/ml) (range)	234.6 (3.2‐1381.2)	221.9 (4.8‐762.8)	.254^a^
Liver cancer			
HCC	1007 (98.3)	993 (98.7)	.345^b^
ICC	17 (1.7)	13 (1.3)	
Median maximal tumor diameter (cm) (range)	2.8 (0.7‐9.8)	2.9 (0.8‐11.2)	.188^a^
No. of tumours	1670	1578	.139^b^
Single	578 (78.8)	552 (58.8)	
Multiple	446 (21.2)	154 (41.2)	
Subcapsular			
Presence	177 (39.6)	181 (40.3)	.445^b^
Absence	847 (60.4)	825 (59.7)	
Ablation modality			
RFA	162 (15.8)	167 (16.6)	.801^b^
MWA	862 (84.2)	839 (83.4)	
Ablation sessions^c^, ^d^	1945	1872	.578^b^
1	834 (80.8)	801 (81.6)	
>1	190 (19.2)	205 (18.4)	
Median platelet counts (×10^9^) (range)	109 (67‐459)	110 (75‐751)	.562^a^
Mean INR ± SD (range)	1.13 ± 0.21 (0.87‐1.38)	1.15 ± 0.32 (0.89‐1.49)	.898^a^
Seeding^c^	15/1024 (0.9)	9/1006 (0.8)	.867^b^
Technique effectiveness	1000/1024 (97.6)	985/1006 (97.9)	.762^b^
Follow‐up (y)			
Median	25.6	27.0	.787^a^
Range	4.3‐90.2	6.2‐91.3	

Note

Except where indicated, data are numbers of patients. Data in parentheses are percentages and were calculated by using the total number of patients in each group as the denominator. SD = standard deviation. *P* < .05 indicated a significant difference.

Abbreviations: HCC, hepatocellular carcinoma; ICC, intrahepatic cholangiocarcinoma; MWA, microwave ablation; RFA, radiofrequency ablation; HBV, hepatitis B virus; CTP, Child‐Turcotte‐Pugh; AFP, α‐fetoprotein; AST, aspartate aminotransferase; ALT, alanine aminotransferase; INR, international normalized.

^a^ Student *t* test.

^b^ Pearson *χ* ^2^ test.

^c^ Data in parentheses are percentages.

^d^ Data are the number of treatments.

**TABLE 2:** Characteristics of the 15 patients who had seeding after thermal ablation

No	Age/sex	TS	Size (cm)	Number	TA	DD	SL	ST (mo)	STM	OS (mo)
1	65/M	HCC	4.5	1	B	M	AM	17.7	MWA	36.8
2	58/M	HCC	6.4	1	B	M	AM	4.2	MWA	74.2
3	67/M	HCC	3.5	1	B	M	AM	17.5	MWA	52.6
4	59/M	ICC	6.3	1	B	H	AM	6.3	RFA	14.7
5	48/M	ICC	2.7	2	B	M	AM	22.1	RFA	37.5
6	61/M	HCC	4.9	1	B	H	TM	12.0	SR	32.4
7	71/M	HCC	4.0	1	C	M	AM	30.5	MWA	54.2
8	66/M	HCC	3.7	1	B	M	AM	37.9	MWA	49.0
9	41/M	HCC	2.9	1	B	M	TM	34.3	MWA	44.7
10	47/F	HCC	3.3	2	B	H	AM	12.2	MWA	53.5
11	72/M	HCC	4.7	1	B	H	AM	15.6	SR	22.9
12	78/M	HCC	2.0	1	B	H	AM	24.0	MWA	35.6
13	51/M	ICC	2.3	1	B	H	TM	30.8	MWA	43.2
14	48/M	HCC	2.5	1	C	M	TM	17.8	MWA	67.3
15	61/M	HCC	2.5	1	B	H	TM	28.0	MWA	58.9

Abbreviations: TS, Tumor type; TA, Type of hepatitis; B, hepatitis B virus; C, hepatitis C virus; DD, Differentiated degree; M, Middle; H, High; SL, Seeding location; ST, Seeding time; STM, Seeding treatment modality; OS, Overall survival; HCC, Hepatocellular carcinoma; ICC, Intrahepatic cholangiocarcinoma; AM, Abdominal wall; TM, Thoracic wall; MWA, Microwave ablation; RFA, Radiofrequency ablation; SR, Surgical resection.

### 3.2 Cumulative rate of seeding

**FIGURE 2:**
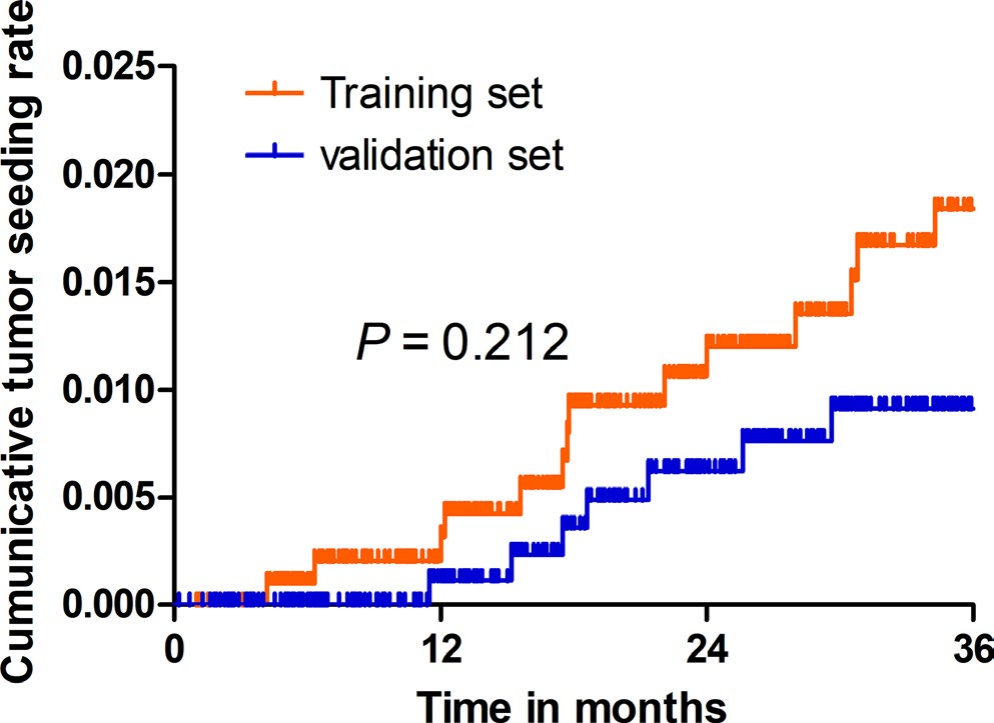
Kaplan‐Meier curves show that ITAS is comparable between the training data set and the validation data set

In the training set, patients with liver malignancy who had undergone PTA showed a 1‐, 2‐ and 3‐year cumulative seeding rate of 0.3%, 1.2% and 1.8%, respectively. In the validation set, patients with liver malignancy who had undergone PTA showed a 1‐, 2‐ and 3‐year cumulative seeding rate of 0.1%, 0.6% and 0.9%, respectively. No significant differences were found between the training and validation sets (*P* = .212) (Figure 2).

### 3.3 Risk factors for seeding in the training set

Univariate and multivariate analyses were used to evaluate fourteen potential risk factors (age, gender, comorbidities, etiology, cirrhosis, CTP grade, α‐fetoprotein [AFP], tumor size, number, subcapsular location, ablation modality, international normalized ratio [INR], platelet and sessions) for seeding. In the univariate analyses, tumor size, tumor number, subcapsular location, AFP, INR and sessions were found to be significantly associated with the presence of seeding after PTA in the training data set (Table 3). In the multivariate analyses, larger tumor size (>5 cm) (*P* = .005; 95% confidence interval (CI): 1.964‐17.226; hazard ratio (HR): 5.087), subcapsular location (*P* < .001; 95% CI: 2.188‐14.437; HR: 6.520), AFP level of >20 ng/mL (*P* = .017; 95% CI: 1.752‐4.262; HR: 2.997) and INR of <1.01 (*P* = .023; 95% CI: 0.126‐0.856; HR: 0.329) were found to be independent risk factors associated with seeding after PTA (Table 4).

**TABLE 3:** Univariate analysis for seeding after thermal ablation for liver malignancy

Factors	No. of patients	Univariate analysis	
		HR (95% CI)	*P* value*
Age (y)			
<65	412	1.908 (0.948, 3.838)	.270
≥65	612		
Gender			
Male	837	0.839 (0.462, 1.524)	.565
Female	187		
Comorbidities			
Absence	410	2.129 (0.651, 6.961)	.211
Presence	614		
Etiology			
HBV	645	3.782 (0.703, 5.141)	.516
Other	379		
Cirrhosis			
Absence	94	1.404 (0.508, 3.875)	.513
Presence	930		
Tumor size (cm)			
<5	478	2.072 (1.250, 3.436)	.005
5‐12	546		
No. of tumors			
Single	578	5.627 (2.992, 10.581)	.043
Multiple	446		
Subcapsular			
Absence	177	3.110 (1.097, 8.817)	.003
Presence	847		
Ablation modality			
RFA	373	1.227 (0.907, 1.661)	.185
MWA	167		
AFP			
≤20	285	1.428 (0.732, 2.788)	.026
>20	255		
INR			
≤1.1	316	2.012 (1.582, 5.760)	.007
>1.1	708		
CTP grade			
A	976	1.110 (0.497, 3.817)	.233
B	48		
Ablation sessions			
1	823	3.245(1.432, 5.212)	.033
>1	201		

Note

Data in parentheses are 95% confidence intervals.

Abbreviations: HR, hazard ratio; CI, confidence intervals; HBV, hepatitis B virus; CTP, Child‐Turcotte‐Pugh; AFP, α‐fetoprotein; INR, international normalized ratio.

* *P* values were determined with Cox proportional hazards regression models. *P* < .05 indicated a significant difference.

**TABLE 4:** Multivariate analysis of seeding after thermal ablation with Cox proportional hazards model

Variable	β level	SE	Wald	*P* value	HR	95% CI	
						Upper	Lower
Tumor diameter	1.568	0.537	10.850	.005	5.087	1.964	17.226
Subcapsular	1.854	0.483	13.590	<.001	6.520	2.188	14.437
AFP	1.308	0.575	3.940	.017	2.977	1.757	4.262
INR	‐1.113	0.489	3.357	.023	0.329	0.126	0.856

Abbreviations: MWA, microwave ablation; HR, hazard ratio; CI, confidence intervals; AFP:α‐fetoprotein; INR, international normalized ratio.

### 3.4 Construction of the nomogram

**FIGURE 3:**
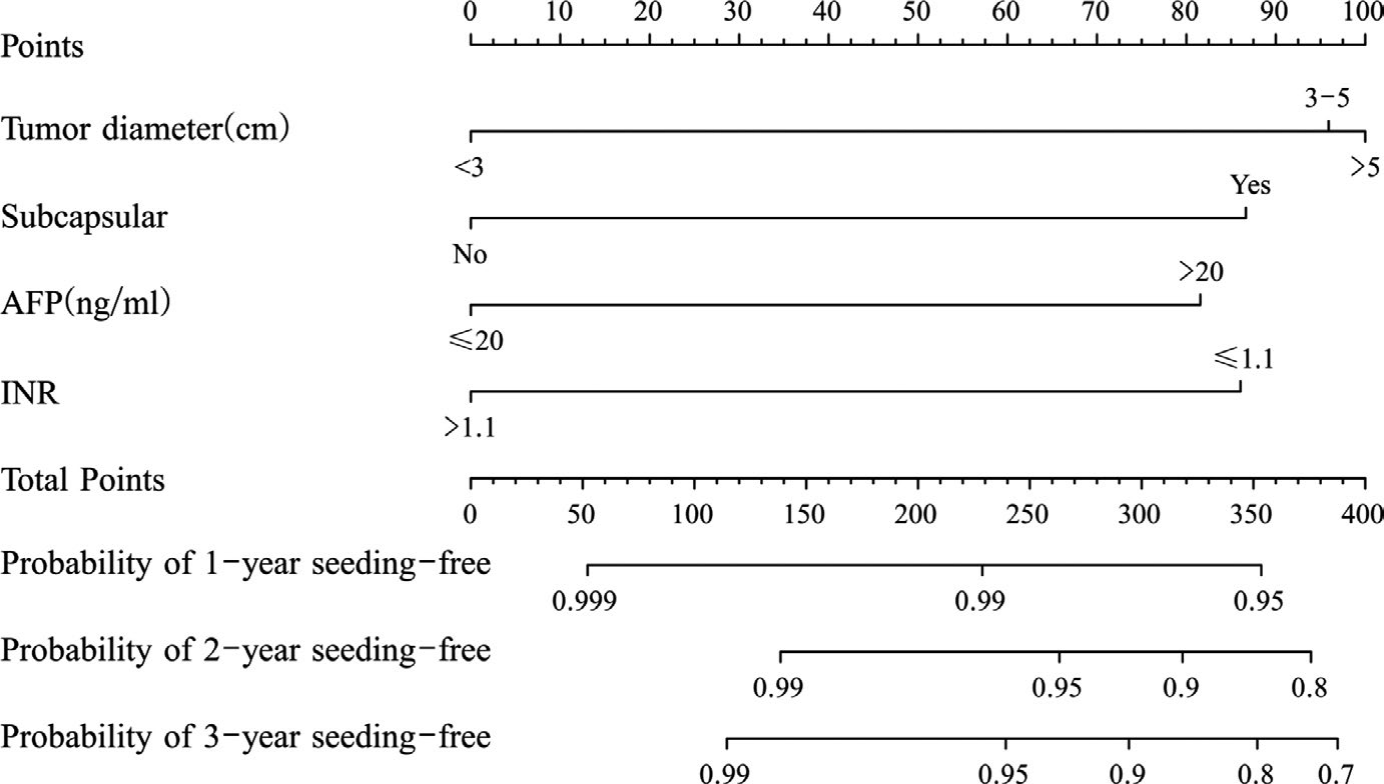
The nomogram was developed in the training data set, with tumor size, subcapsular, AFP level, and INR

**FIGURE 4:**
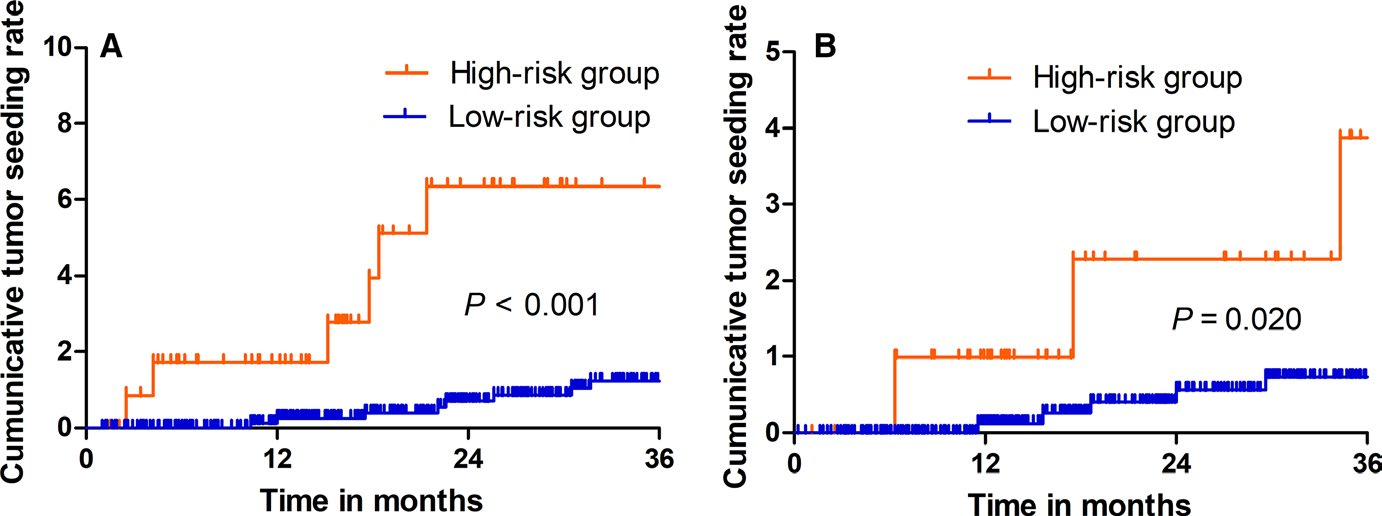
The cumulative seeding rate stratified by risk score of nomogram was then used to plot Kaplan‐Meier curves. (A) The cumulative seeding rate in the high‐risk group was higher than that in the low‐risk group in training sets. (B) The cumulative seeding rate in the high‐risk group was higher than that in the low‐risk group in validation sets

Tumor size, subcapsular location, AFP and INR levels in data of the training set were used to construct the nomogram shown in Figure 3. The nomogram was obtained using the four preidentified prognostic risk factors, and a predetermined score was allocated to each component. For each patient, the sum of points was used to predict the risk of seeding at 1, 2 and 3 years postablation. Then, Kaplan‐Meier curves were plotted for the data of the validation and training sets (Figure 4) using the cumulative seeding rate stratified by the risk score of the nomogram. The cumulative seeding rate at 1, 2 and 3 years was 0.1%, 0.7% and 1.2%, respectively, for the low‐risk group and 1.7%, 6.3% and 6.3%, respectively, for the high‐risk group, showing significant statistical differences (*P* < .001) in the training set. The cumulative seeding rate at 1, 2 and 3 years was 1.0%, 2.3% and 3.9%, respectively, for the high‐risk group, and 0.1%, 0.4% and 0.7%, respectively, for the low‐risk group, showing significant statistical differences (*P* = .020) in the validation set.

### 3.5 Validation and predictive accuracy of the nomogram

**FIGURE 5:**
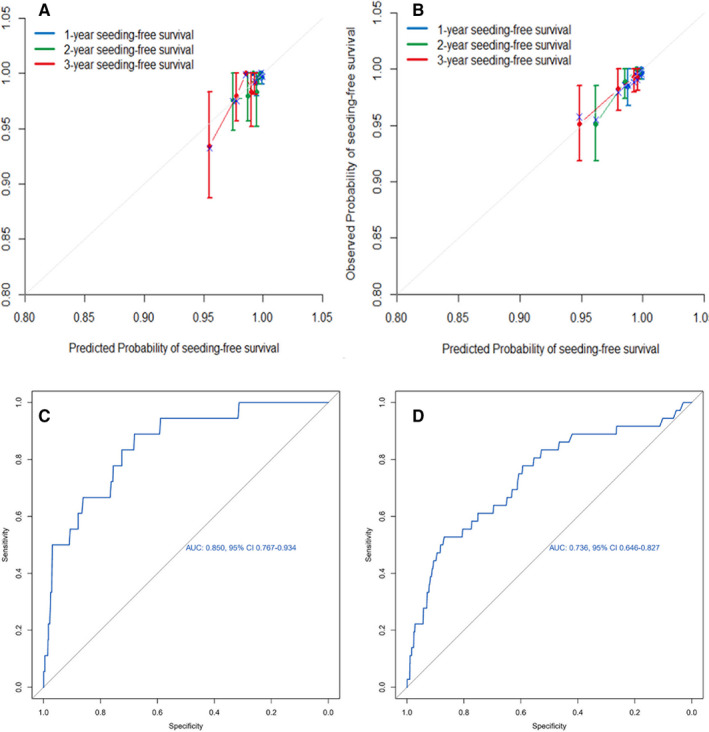
Calibration curves of the nomogram at 1‐,2‐, 3‐years cumulative seeding rate in (A) the training data set and (B) the validation data set shows good correlation between assessed and observed outcomes. Calibration curves were close to 45° line. Predictive accuracy for seeding risk after PTA in the nomogram. (C) An AUC value of 1.850 is shown in the training data set; (D) An AUC value of 0.736 is shown in the validation data set

In the training set, the probability of seeding at 1, 2 and 3 years after PTA in the calibration plot showed fair agreement with the prediction made using actual observations and the nomogram (Figure 5A). Seeding prediction showed a C‐index of 0.722 (95% CI: 0.661‐0.883). In the validation set, the probability of seeding at 1, 2 and 3 years after PTA in the calibration plot showed fair agreement with the prediction made using actual observations and the nomogram (Figure 5B). Seeding prediction showed a C‐index of 0.706 (95% CI: 0.546‐0.866). The discriminative ability of the nomogram was assessed using the area under the curve (AUC), and the AUC for the training set and validation set were 0.850 (95% CI: 0.767‐0.934) and 0.736 (95% CI: 0.640‐0.827), respectively (Figure 5C,D).

## 4 DISCUSSION

Although PTA is considered a minimally invasive and relatively safe technique, tumor seeding on the chest or abdominal wall remains fail to be obviated. Therefore, the transformation between the benefits and risks must be considered and a clearer understanding of the pertinent complications which may occur after PTA is the key to subsequent successful treatments. We report the results obtained in 1024 liver malignancy patients with 1670 lesions treated by PTA over a 9‐year period in training set. The rate of neoplastic seeding was 0.89% for per tumor when major application (84.2%) of MWA was found. Although the advantages of MWA include a higher intratumoral temperature, short operation time as well as less dependence on electrical conductivities, and MWA with a lower seeding rate comparing with RFA in previous reports,^28^, ^29^, ^30^ ablation modality (RFA or MWA) is not the risk factor in our study.

A large number of evidences support the concept that seeding risk after PTA is driven by a complex interplay between unmodifiable patient‐ and tumor‐related factors and modifiable ablation‐related technical factors. The qualitative factors of the seeding incidence rate are determined by tumor size, pathological differentiation, and tumor location, while the quantitative factors depend on ablation parameters including ablation power, ablation time, insertion, and session. These factors together determine the seeding risk after PTA. Therefore, both the factors should be considered for the cause of seeding. In this study, tumor size, subcapsular, and AFP were the risk factors for seeding after PTA, which was consistent with the previous reports.^15^, ^16^, ^17^ Whereas INR needs to be further verified in a larger sample cohort.

The seeding mechanism currently has two views, as follows: (a) when the biopsy needle or ablation applicator is withdrawn, viable tumor cells adhered to these implementations fall off on the chest wall or abdominal wall; (b) due to sudden intratumoral hyper‐pressure during ablation procedure, tumor cells dash on the thoracoabdominal wall along the needle track.^12^, ^14^, ^31^ However, clinicopathological parameters causing the seeding risk after PTA need to be further explored. A large number of evidences support the concept that seeding risk after PTA is driven by a complex interplay between unmodifiable patient‐ and tumor‐related factors and modifiable ablation‐related technical factors. The quantitative factors of the seeding incidence rate are determined by tumor type, size, pathological differentiation, and location, while the qualitative factors depend on ablation parameters (ie ablation power, ablation time, insertion and session) and needle tract ablation. Therefore, both the types of factors should be considered for the cause of seeding.

Here, we developed and validated a clinicopathological‐based nomogram to improve the prediction of seeding risk. The nomogram incorporated four risk factors from the conventional clinicopathological characteristics of patients with liver malignancy underwent PTA, including tumor size, subcapsular, and biopsy. We developed a repeated and easy‐to‐use, economical nomogram that facilitated the individualized prediction of the risk of seeding after PTA. The aim of our study was to maximize the clinical relevance of the nomogram by increasing applicability in preablation patient counseling as follows: an interventional radiologist can estimate effectively the seeding risk after PTA and develop a preoperative ablation program to reduce the likelihood of the event, when using the nomogram. Once a PTC nodule in diameter >5 cm is encountered, we should pay attention to the biopsy procedure before ablation. If there are two typical imaging findings and alpha‐fetoprotein elevation, biopsy should be avoided, and when the tumor is under the liver capsule, when punctured, we should do our best to pass the normal liver parenchyma and ablate the needle in the process of needle withdrawal.

We observed that the nomogram was highly discriminating in both the training dataset and the external validation dataset. The novel nomogram was then validated by assessing a large, contemporary population of patients with liver malignancy treated by PTA (designated as the external validation data set). The calibration plot related to nomogram for the seeding risk had a C‐index of 0.722 (95% CI: 0.661‐0.883), which indicates good agreement between observed and predicted probabilities. Moreover, the AUC of 0.844 (95% CI: 0.661‐0.883) confirmed the benefit of the nomogram. One possible use for this nomogram is in patient selection for inclusion into clinical trials based on their individual risk of seeding postablation. Randomization may be further stratified based on either a two‐ or three‐risk grouping. To high‐risk seeding patients, MWA is a safe and effective treatment method, which achieve satisfactory survival outcome compared with surgical resection in our previous study.^32^

Our study has several limitations indeed. Firstly, this study is a retrospective one. A prospectively designed cohort study would allow greater elimination of bias in assessment of the various risk factors. Secondly, the long time span of this study may allow PTA operators to improve their technique as the study goes by and thereby affect seeding appearance depending on their time of enrollment in the study; Thirdly, when patients with multiple HCCs including one subcapsular tumor, this phenomenon may cause confounding bias.

In conclusion, the development of nomogram has been found to be useful in risk stratification of seeding in patients with PLC after PTA. Based on this risk visual tool, we can choose a reasonable and effective treatment plan according to the clinical characteristics of these PLC patient to reduce the seeding risk. Nevertheless, this novel scoring system will require further validation by more sample before widespread implementation in clinical practice.

## CONFLICT OF INTEREST

The authors declare no conflict of interest.

## AUTHOR CONTRIBUTIONS

CA was involved in conceptualization. ZMH was involved in methodology. TQZ was involved in software and investigation. CA and JYN were involved in validation. MXZ was involved in formal analysis. YQJ was involved in resources and involved in data curation. CA was involved in writing—original draft preparation. JHH was involved in writing—review and editing and funding acquisition.

Guarantor name, Jinhua Huang.

## ETHICS APPROVAL AND CONSENT TO PARTICIPATE

The ethics committee board of the Sun Yat‐sen University Cancer Center, approved the use of patients with PLC after PTA for this study.

## Data Availability

Please contact the corresponding author for all data requests.
